# Epidemiology of Human Metapneumovirus Infection—A Systematic Review and Meta‐Analysis

**DOI:** 10.1002/hsr2.73234

**Published:** 2026-09-13

**Authors:** Sumaiya Tasnim, Sajida Sultana Shammy, Najmul Haider, Md Jamal Uddin

**Affiliations:** ^1^ Department of Statistics Shahjalal University of Science and Technology Sylhet Bangladesh; ^2^ School of Life Sciences, Faculty of Natural Sciences Keele University Staffordshire UK; ^3^ Department of Computer Science and Engineering Daffodil International University Dhaka Bangladesh

**Keywords:** community‐acquired pneumonia, human metapneumovirus, meta‐analysis, respiratory infections, systematic review

## Abstract

**Background:**

Human metapneumovirus (hMPV) is a globally circulating respiratory pathogen. The objective of this systematic review is to evaluate the prevalence, case fatality rate, age‐related severity, and seasonality of hMPV in the group of Acute Respiratory Infections (ARIs) and Community‐Acquired Pneumonia (CAP).

**Methods:**

We conducted a systematic review and meta‐analysis of articles on hMPV published from January 2001 to June 2025. Following PRISMA 2020 guidelines, we systematically searched PubMed and ScienceDirect for eligible articles using strict inclusion and exclusion criteria. Random‐effects meta‐analysis was performed to estimate pooled prevalence, and subgroup analyses were conducted by study group, geographic region, COVID‐19 period, and age group.

**Results:**

One hundred one studies met the inclusion criteria, of which 96 contributed to the meta‐analysis. The pooled global prevalence of hMPV was 6% (95% CI: 5%–7%). Prevalence estimates were highest in patients with respiratory tract infections (RTIs) patients (6%) and in South America (10%). Age‐stratified analysis showed a high prevalence in children under 5 years (7%). Seroprevalence studies demonstrated low hMPV antibody prevalence in young children less than 1 year. The case fatality rate ranged from 2.5% in elderly populations to 4.4% in severe pediatric cases. hMPV mainly affects children under 2 years, with a smaller burden in older adults. Seasonal peaks were typically observed in late winter to early spring.

**Conclusion:**

hMPV remains an important cause of respiratory disease worldwide, with a disproportionate burden on children. The significant heterogeneity across studies and evidence of publication bias highlight the need for standardized surveillance.

## 1 Background

Human metapneumovirus (hMPV), a member of the genus *Metapneumovirus* within the subfamily *Pneumovirinae* and family *Paramyxoviridae*, was first detected in the Netherlands in 2001 in pediatric patients with respiratory infections. However, retrospective serological analyses indicate that the virus has been infecting humans for over five decades, suggesting it is not a newly emerging pathogen [1, 2]. Since its identification, hMPV infections have been documented globally across all age groups, including young children, the elderly, and those with weakened immune systems [3, 4]. hMPV has a worldwide distribution and infects nearly all children by age 5–10 [2]. It is a major viral cause of acute respiratory tract infections (RTIs), ranging from mild cold‐like illness to severe conditions such as bronchiolitis and pneumonia, with significant impact on global morbidity and mortality, especially among vulnerable populations during colder months [4, 5]. For instance, research from Cordoba, Argentina, reported a prevalence of 20.3% of hMPV among patients with severe acute respiratory infections (ARIs), emphasizing its clinical importance [6]. Another study found that the late‐life (elderly) age group had a 10%–15% prevalence of HMPV; however, many patients were co‐infected with other viruses, such as influenza and RSV [7]. Gaining insights into the virus's epidemiological patterns, including prevalence rates, mortality risk, and age‐based susceptibility, is crucial for informing public health strategies and preventive measures.

Seroprevalence data play a crucial role in epidemiology by revealing the extent of prior viral exposure, the level of immunity within a population, and the likelihood of future outbreaks [8]. Research indicates hMPV is a significant cause of respiratory illness in children, with nearly universal seropositivity—close to 100%—by the age of five [3, 9, 10]. This high seroprevalence highlights the virus's widespread transmission in early childhood [11]. Despite early exposure, individuals across all age groups can experience reinfections, implying that immunity acquired from initial infection is not long‐lasting and that hMPV may remain a persistent health concern throughout life [4, 12]. In a Thai study, 99.7% of children showed evidence of prior hMPV infection, with a 4.9% reinfection rate [13]. Comparisons with respiratory syncytial virus (RSV) indicate that hMPV seroprevalence is generally lower in children aged 6 months to 6 years [14]. Neutralizing antibodies remain high across all age groups, with a slight decrease in individuals over 69 years [15]. These findings highlight the widespread transmission of hMPV in early childhood and its potential for causing significant outbreaks in older children and adults.

The case fatality rate (CFR) is a critical measure for evaluating the impact of a disease, as it indicates the proportion of diagnosed individuals who die from the illness within a specific population [16]. As an indicator of disease severity, CFR helps identify vulnerable groups and supports the development of targeted public health interventions [17]. A systematic review and meta‐analysis of adults found pooled hospital mortality near 10% and ICU mortality about 23% across heterogeneous studies, highlighting substantial variability by study and patient mix [18]. Pediatric data show much lower fatality in routine hospitalized children, with one large pediatric cohort reporting 2 deaths among 815 hospitalized children (≈0.2%) [19]. Reported case fatality in single‐center adult series and outbreak investigations is higher, with some institutional series and outbreaks documenting in‐hospital fatality in the range of ~20%–38% in small or high‐risk samples [20, 21]. This makes CFR an important metric for assessing both the clinical severity of hMPV and its associated healthcare burden [22].

Recent post‐COVID‐19 outbreaks have intensified global interest in the epidemiological dynamics of human metapneumovirus (hMPV). In late 2024, hMPV emerged as a more frequently detected respiratory virus than COVID‐19, rhinovirus, or adenovirus in China, accounting for 6.2% of positive respiratory specimens and 5.4% of related hospitalizations [23, 24]. In early 2025, India identified a localized outbreak involving five infant cases, in which two cases in Bengaluru, one in Ahmedabad, and two in Tamil Nadu [25]. The clinical characteristics, epidemiological trends, and seasonal fluctuations of hMPV have gained more focus due to severe outbreaks, particularly the recent increases in China and Malaysia following the COVID‐19 pandemic [26]. These outbreaks underscore the importance of regional and global surveillance, diagnostic preparedness, and preventive strategies. Moreover, the lack of specific antiviral treatments or licensed vaccines against hMPV highlights the urgency for further epidemiological research.

The present study aimed to conduct a systematic review and meta‐analysis of published studies on human metapneumovirus (hMPV) outbreaks to evaluate the prevalence, CFR age‐related severity, and seasonality of hMPV. The quantitate synthesis will perform with sufficient database otherwise the outcomes will summarize descriptively.

## 2 Methods

The PRISMA 2020 guidelines were followed for reporting the findings of the Systematic Review and Meta‐Analysis. The protocol has been registered with the International Prospective Register (PROSPERO), and the registration ID is CRD420251044010. This systematic review considered the 2020 Reporting Items for Systematic Reviews and Meta‐Analysis (PRISMA) [27] guidelines during its execution. The PRISMA checklist for abstract (Supporting Information S1: Appendix Table S1) and manuscript (Supporting Information S1: Appendix Table S2) are attached in the Supplementary files.

### 2.1 Search Strategy

We conducted a systematic literature search in PubMed and ScienceDirect between January 1, 2025 and June 30, 2025, including articles published between January 1, 2001 and April 30, 2025. PubMed provides comprehensive coverage of biomedical and health‐related literature, while ScienceDirect also included relevant publications. We searched articles with the basic search terms: (“Human Metapneumovirus” OR “HMPV” OR “hMPV”) AND (“case fatality rate” OR “case fatality ratio”, OR “CFR” OR “basic reproduction number”, OR “R0”, OR “seroprevalence” OR “prevalence”) according to the database (Supporting Information S1: Appendix Table S6). To structure the search strategy, the terms were grouped into three categories: (1) hMPV AND prevalence/seroprevalence, (2) hMPV AND CFR, and (3) hMPV AND R0. Through the manual screening, the consistency has been ensured with specialists in the field of analysis. All the articles were stored, screened, and duplicates were excluded by Rayyan software (www.rayyan.com).

### 2.2 Inclusion and Exclusion Criteria

Articles were selected that reported the prevalence of hMPV considering three particular groups of patients: laboratory‐tested patients for hMPV, Acute Respiratory Infections (ARIs), and Community‐Acquired Pneumonia (CAP). The study included patients with laboratory‐confirmed hMPV. Studies that measured seropositivity in co‐infected or reinfected patients were excluded. In total, 101 studies were conducted with an adequate sample size, included all age groups, and were published in English and in any country or region worldwide. Exclusion criteria included preprints, review papers, editorials, case reports, letters to the editor, short communications, and studies focusing on youngsters or pregnant women (Supporting Information S1: Appendix Table S3).

### 2.3 Data Extraction

Two investigators (S.T. and S.S.) independently reviewed full‐text publications for inclusion after removing duplicates and screening titles and abstracts based on eligibility criteria. The co‐authors helped resolve any disagreements. Data from qualifying studies were extracted using a standardized form. Include publication facts (title, first author, year, and journal name), study design and population (country, study period, and sample size), participant characteristics, and key findings for each paper (prevalence, seroprevalence, vulnerable age, and season of high prevalence). The prevalent age and seasonal information were reported from the selected studies, if any study reported such information.

### 2.4 Critical Appraisal and Publication Bias

The authors independently evaluated each selected study using the Joanna Briggs Institute (JBI) [28] criteria for quality assessment of the included studies (Supporting Information S1: Appendix Table S5). A funnel plot was employed to assess publication bias, and Egger's test (*p* < 0.05) indicated small‐study effects. Sensitivity analysis was conducted to ensure the robustness of the study.

### 2.5 Statistical Analysis

**Figure 1:**
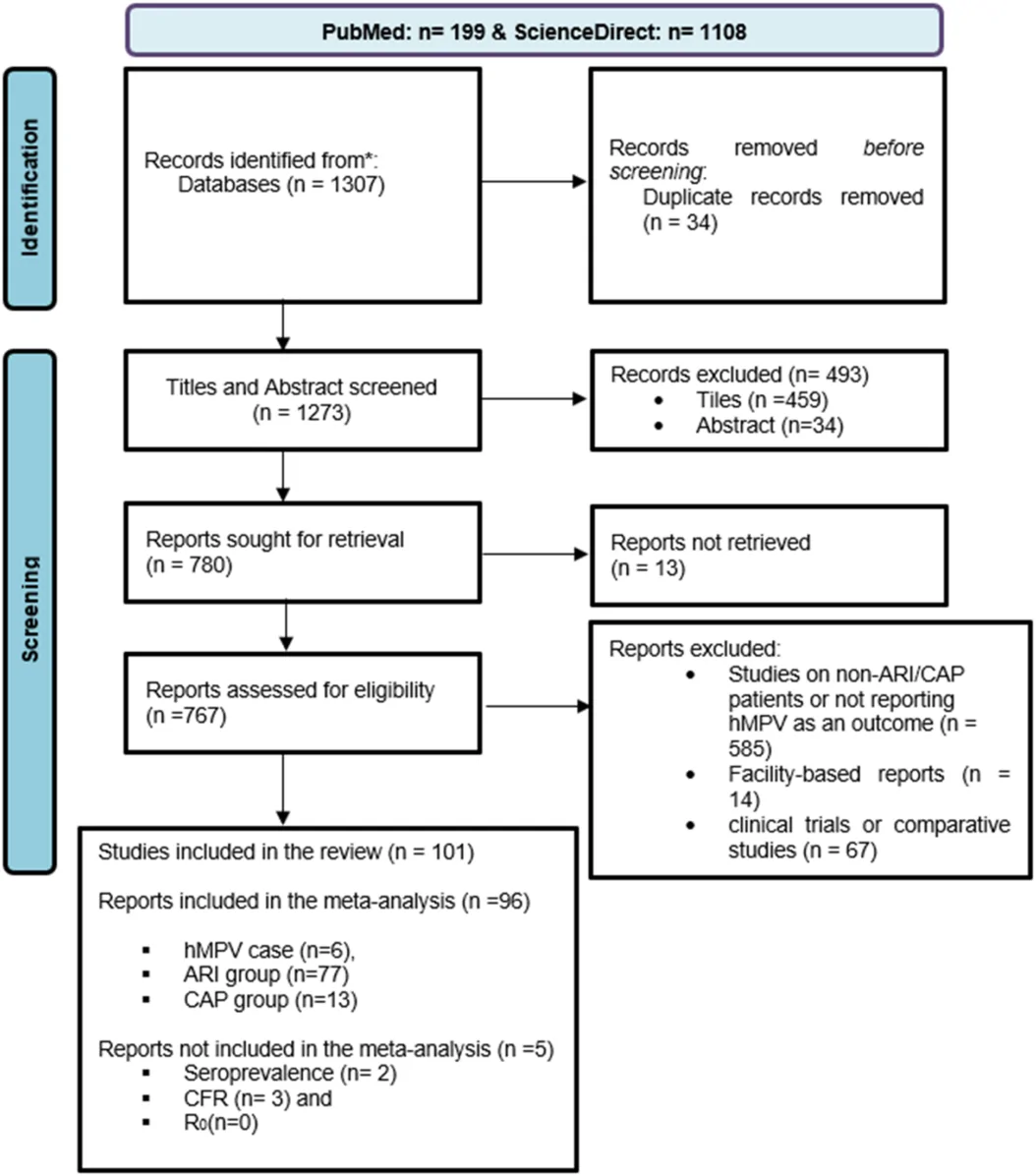
Flow chart for the studies included in the systematic review and meta‐analysis.

The primary goal of this meta‐analysis was to determine the effect size, such as the combined prevalence, as measured by the proportion of hMPV in laboratory‐tested patients of hMPV or ARIs, also the proportion of hMPV in CAP patients. The pooled prevalence was calculated using the effect size method, which aggregates the proportion of all studies to get the overall effect. However, there were not enough studies to conduct a meta‐analysis for, CFR, basic reproduction rate (*R*_0_), seroprevalence, and seasonality. Thus, meta‐analysis was only performed for prevalence and other estimates were reported descriptively. The study explored the aggregate prevalence of hMPV in ARIs and CAP groups separately. We analyzed study variability using the *I*‐squared statistic and Cochran's *Q* statistic, as well as the corresponding *p*‐value showing heterogeneity. A random‐effects model was used to obtain aggregate estimates, which were then reported as proportions with 95% confidence intervals attached. Meta‐analyses were conducted using STATA 16.0 software (STATA Corp). Figure 1 shows the article selection and screening process. To further investigate the study heterogeneity, univariate and multivariable random‐effects meta‐regression were performed using study group, age group, continent, COVID‐19 period, publication year, and diagnostic method.

## 3 Results

A total of 101 studies met the inclusion criteria. Among these, 96 reported hMPV prevalence, 2 reported seroprevalence, and 3 reported CFR. Of the included studies, 30 identified age‐related vulnerability to hMPV infection, and 42 described its seasonal distribution. Based on study populations, 6 studies included individuals specifically tested for hMPV, 77 focused on patients with acute respiratory infections (ARIs), and 13 examined community‐acquired pneumonia (CAP) cases.

Due to the limited number of studies reporting seroprevalence (*n* = 2) and case fatality rate (CFR; *n* = 3), these outcomes were summarized descriptively rather than included in the meta‐analysis. Consequently, the meta‐analysis was conducted exclusively on hMPV prevalence, incorporating subgroup analyses based on data from the remaining 96 studies.

### 3.1 Results of Review

#### 3.1.1 Estimate of Seroprevalence

Two studies have assessed the seroprevalence or seroconversion of (hMPV) infection in different populations and age groups with a prevalence of 43% and 18.7% respectively.

In a study from Japan, serum samples were tested involving 100 children aged 1 month to 5 years. Among children over 4 months of age (*n* = 91), the seropositivity for hMPV was 43%. The lowest hMPV seropositivity was observed in children aged 4 months to 1 year (11%), whereas 67% of infants younger than 4 months were seropositive, likely due to maternal antibodies.

A second Japanese study enrolled 337 adolescents and adults (≥ 16 years) with acute lower respiratory tract infections between 2017 and 2019, using paired sera to determine hMPV seroconversion via focus reduction neutralization assay. Overall, 18.7% (63/337; 95% CI: 14.9%–23.2%) of patients demonstrated seroconversion, with annual rates of 7.0% in 2017, 23.6% in 2018, and 16.0% in 2019. Among pneumonia cases in adults, hMPV accounted for 19.4% (56/288) of infections. These results indicate that hMPV is widely circulating in both pediatric and adult populations, with low antibody prevalence in young children after maternal antibody decline, and measurable annual seroconversion in adults during seasonal epidemics.

#### 3.1.2 Case Fatality Rate of hMPV

The reviewed evidence from three studies provides insight into the case fatality rate (CFR) associated with human metapneumovirus (hMPV) infection across different populations and settings. A prospective cohort study conducted in seven African and Asian countries among children aged 1–59 months hospitalized with severe pneumonia reported a fatality rate of 3.9% among hMPV‐positive cases. A retrospective case‐control study of a nursing home outbreak in France found a fatality rate of 2.5% among older adults, reflecting increased vulnerability in elderly populations. In the United Kingdom, a retrospective analysis of pediatric intensive care admissions over 9 years reported a CFR of 4.4%, representing the most severe cases. Overall, these three studies suggest that hMPV‐related mortality is generally low but can be higher in high‐risk groups such as young children, older adults, and critically ill patients.

#### 3.1.3 Age and hMPV

A review of the included studies revealed that hMPV infection predominantly affects younger age groups, with the highest susceptibility observed in infants and young children. Several studies identified infants under 1 year of age as the most vulnerable, while others extended this high‐risk category to children up to 24 months of age. Broader definitions of vulnerability included children under 3 years, under 4 years, or under 5 years. Some investigations specified narrower peaks, such as 7–12 months, 6–12 months, or 18–24 months, highlighting variation in age distribution patterns across study settings.

In addition to pediatric cases, a smaller number of studies reported a substantial disease burden among older adults, particularly those aged ≥ 51 years or ≥ 65 years, suggesting a possible bimodal distribution of severe hMPV cases. Collectively, the literature indicates that the most vulnerable groups for hMPV infection are infants and young children (especially those under 2 years of age) and, to a lesser extent, older adults.

#### 3.1.4 Seasonality (Hemisphere‐Specific)

Most of the reported studies were conducted in the Northern Hemisphere (*n* = 38), particularly in Asia (China, Japan, India, Taiwan, Sri Lanka, Jordan, Cambodia, and Israel) and Europe (United Kingdom, France, Spain, Switzerland, Belgium, and Scotland) (Supporting Information S1: Appendix Table S4). In the Northern Hemisphere, human metapneumovirus (hMPV) infections typically peak during winter and spring (December–May). Some Asian countries (e.g., China, Japan, India, and Taiwan) also reported secondary peaks extending into early summer (June), while tropical regions (Sri Lanka and Cambodia) showed seasonality linked to the rainy/monsoon seasons (April–October).

Only a few studies (*n* = 4) were conducted in the Southern Hemisphere, including those from Argentina and South Africa. In the Southern Hemisphere (Argentina and South Africa), hMPV infections peaked during the cooler months, spanning late winter to spring (June–November). Overall, the dominant seasonality pattern is winter–spring across both hemispheres, with peak months occurring in February–April in the Northern Hemisphere and August–November in the Southern Hemisphere.

### 3.2 Results of Meta‐Analysis

The overall pooled prevalence (Table 1) across all 96 studies was 6% (95% CI: 5%–7%), with significant heterogeneity (*I* ^2^ = 99.81%, Cochran's *Q* = 5440.00, *p* < 0.001). Visual inspection of the funnel plot revealed an asymmetrical distribution of effect sizes, suggesting the potential presence of publication bias. Egger's regression test confirmed small‐study effects (*p* < 0.05), indicating small study effect due to publication bias. Sensitivity analysis did not show any substantial changes to the pooled estimates, indicating robustness of the findings. The meta‐regression findings (Supporting Information S1: Appendix Table S7) suggested that none of the study variables can significantly explained the between‐study heterogeneity (overall Wald $\chi^{2}=10.79,$ *p* = 0.70). The heterogeneity is likely attributable to other unmeasured factors.

**Table 1:** Effect size and test of heterogeneity for prevalence of hMPV by study group, continent, and COVID‐19 period.

Subgroups	Effect Size (95% CI)	*I* ^2^ Statistics (%)	Cochran's *Q* statistic	No. of study	Test for subgroup variations
Study Group					
hMPV	0.05 (0.02,0.06)	98.24	—	6	3.77 (*p*‐value = 0.15)
RTIs	0.06 (0.05,0.08)	99.84	—	77	
CAP	0.04 (0.02,0.07)	98.74	—	13	
Continent					
Asia	0.06 (0.03,0.09)	99.94	—	45	3.48 (*p*‐value = 0.63)
Africa	0.06 (0.03,0.09)	95.92	—	4	
Australia	0.05 (0.04, 0.06)	0.13	—	2	
Europe	0.05 (0.04,0.06)	98.51	—	23	
North America	0.05 (0.04, 0.07)	91.13	—	15	
South America	0.10 (0.05, 0.15)	98.23	—	7	
COVID‐19 Period					
Before COVID‐19	0.06 (0.05, 0.08)	99.83	—	89	0.36 (*p*‐value = 0.55)
After COVID‐19	0.05 (0.02, 0.08)	97.91	—	7	
Age Group					
Under 5	0.07 (0.04,0.10)	99.60	—	28	5.75 (*p*‐value = 0.06)
Children (< 18 y)	0.07 (0.04, 0.12)	99.83	—	28	
Adult (> = 18 y)	0.04 (0.03,0.05)	99.20	—	40	
*Overall*	0.06 (0.05,0.07)	99.81	5440.00 (*p*‐value = 0.00)	96	

#### 3.2.1 Subgroup Analysis

The prevalence of hMPV was examined through a meta‐analysis that considered four subgroups: Study group or target population group, geographic location, existence of COVID‐19 duration (2020 or later) at the end of data collection, and median population age group.

#### 3.2.2 Study Group

**Figure 2:**
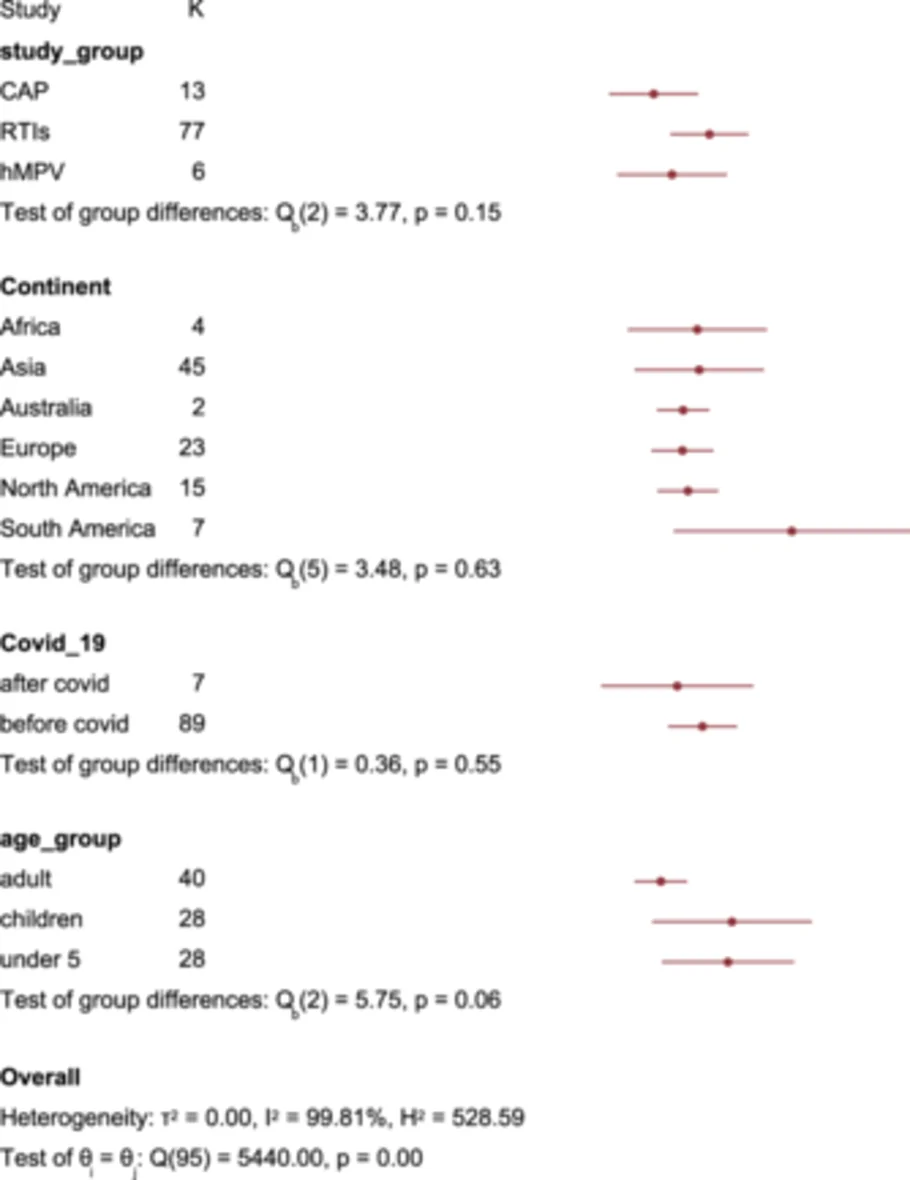
Forest plot of prevalence of hMPV by different subgroups.

**Figure 3:**
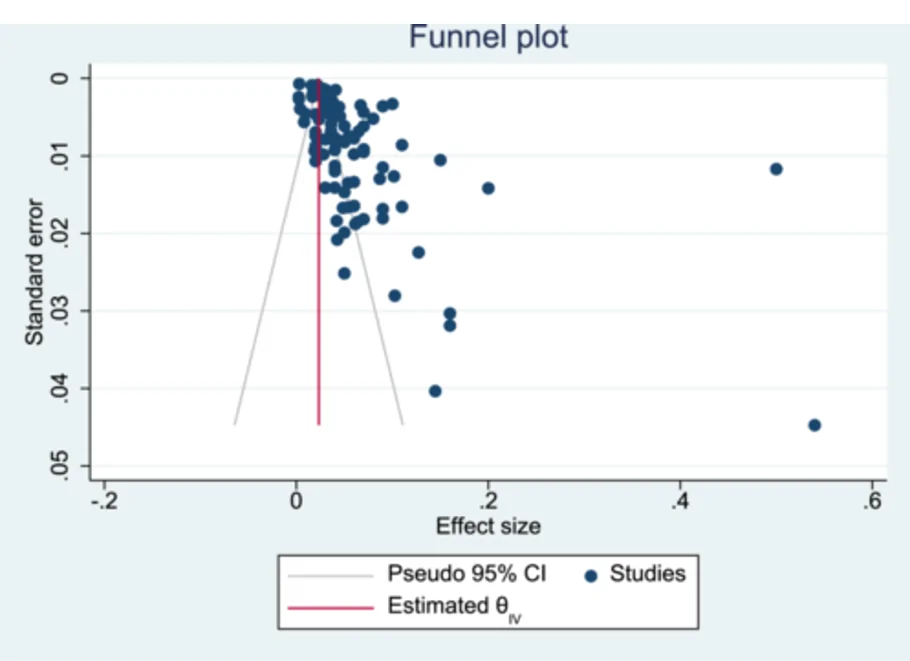
Funnel plot of prevalence of hMPV using random‐effect model.

The estimated pooled prevalence of infection was highest among studies related to respiratory tract infections (RTIs) at 6% (95% CI: 5%–8%), followed by community‐acquired pneumonia (CAP) at 4% (95% CI: 2%–7%), and hMPV studies at 5% (95% CI: 2%–6%). High heterogeneity was observed in all groups (*I* ^2^ > 98%). However, the test for subgroup differences was not statistically significant (Cochran's *Q* = 3.77, *p* = 0.15), suggesting no strong evidence of variation across these study types. Figure 2 shows the effect size with 95% CI corresponding to each subgroup analysis. The funnel plot exhibits slight asymmetry, suggesting potential bias or small‐study effects (Figure 3).

#### 3.2.3 Continent

When stratified by continent, South America showed the highest prevalence estimate at 10% (95% CI: 5%–15%, *n* = 7), whereas Australia reported the lowest at 5% (95% CI: 4%–6%, *n* = 2). Studies from Asia and Africa reported similar pooled estimates at 6% (95% CI: 3%–9%, *n* = 4), followed by Europe at 5% (95% CI: 4%–6%, *n* = 23) and North America at 5% (95% CI: 4%–7%, *n* = 15). Substantial heterogeneity was observed in most regions except Australia (*I* ^2^ = 0.13%). The subgroup difference was not statistically significant (*Q* = 3.48, *p* = 0.63).

#### 3.2.4 COVID‐19 Period

The prevalence was slightly higher in studies conducted before the COVID‐19 pandemic, defined as studies with data collection completed prior to 2020 (6%, 95% CI: 5%–8%, *n* = 89), compared to those studies with data collection ended during or after the onset of the pandemic (5%, 95% CI: 2%–8%, *n* = 7). Both groups showed considerable heterogeneity (*I* ^2^ > 97%), but the difference was not statistically significant (*Q* = 0.36, *p* = 0.55).

#### 3.2.5 Age Group

Studies (*n* = 28) whose participants were only limited to children under the age of five were considered in the “under 5” category. The broader pediatric group comprised studies (*n* = 28) that did not only focus on children under the age of five but also covered a wider range of pediatric ages up to eighteen. The adult population group included studies (*n* = 40) those were 18 years of age or older. All these age groups are mutually exclusive. Age‐stratified analysis indicated the pooled prevalence among the studies that consider only children under 5 and the studies that includes broader children age group (< 18 years) are almost similar. The percentage was 7% (95% CI: 4%–12%, *n* = 28) in the < 18 years group and 7% (95% CI: 4%–10%, *n* = 28) in the under‐5 group. In contrast, studies involving adults (≥ 18 years) showed a lower prevalence of 4% (95% CI: 3%–5%, *n* = 40). The test for subgroup difference approached statistical significance (*Q* = 5.75, *p* = 0.06), suggesting possible age‐related differences in infection prevalence.

## 4 Discussions

Our study determined the pooled estimate of prevalence of hMPV with both Respiratory Tract Infections (RTIs) and Community Acquired Pneumonia (CAP), and the estimate was 6% with South America having the highest prevalence (10%). The hMPV prevalence was identified more in studies that collected the data before the COVID‐19 pandemic. Both review and meta‐analysis findings suggest that infant and young children were more exposed to hMPV than other age groups. The study found that in both Northern and Southern Hemispheres, hMPV infections peaked during the cold season, mostly winter to spring. However, in tropical regions, the incidence was higher in the rainy season.

The serological test gives better results; however, the limited data on seroprevalence and mortality observed in our study are consistent with earlier reviews, which have also noted the scarcity of high‐quality, large‐scale sero‐epidemiological data [29]. Similarly, the limited ability to quantify case fatality or basic reproduction rate outcomes across generalized studies has been identified as a recurring limitation in hMPV literature. This gap reflects a broader need for standardized reporting of clinical outcomes and improved case definitions that capture the full clinical spectrum of hMPV.

A study conducted in China found the incidence of HMPV among patients with acute respiratory disease, ranging from 0.97% (95% CI 0.74–1.20) to 15.88% (95% CI 14.41–17.35). Most of the studies used the RT‐PCR method for the detection of hMPV virus, which is extensively utilized for pathogen detection, but it requires complex instruments and trained workers [30]. Similar to our study, previous studies have suggested that hMPV is a globally circulating pathogen with significant impact, particularly among children and respiratory patients [29, 31]. Recent epidemiological figures predict a 17 percent rise in pediatric hMPV‐related hospitalizations in the first quarter of 2025 compared to the period in 2023 in both the USA and China [32].

hMPV causes 5%–7% of all respiratory infections in children admitted to hospitals [25]. In China, hMPV infection is more prevalent in children under the age of one, with a seasonal peak in the spring [30, 33]. A study suggested that in high‐burden environments, hMPV‐associated severe pediatric pneumonia primarily affected young infants [34]. While earlier reviews have predominantly focused on pediatric groups or specific hospital‐based cohorts, this study incorporates the entire population age groups.

Seasonality has been well described in temperate settings, with peaks typically observed in late winter and early spring [31, 35]. Kuang et al. suggested hMPV rotation is higher throughout November to May [36]. This review also captures studies that ended data curation before and just after the COVID‐19 pandemic period, providing insight into the shifts in viral circulation associated with global public health interventions. Consistent with other respiratory virus trends, hMPV activity showed declining trends for those studies that terminated the data collection during the height of the pandemic, followed by other studies [35]. The possible reason would be that people continued to practice good hygiene habits, more commonly wearing masks in public, and washing their hands. Xuena Xu et al. showed that the zero COVID policy can be a useful technique to assess the actual burden of hMPV after the COVID period [37].

Before the zero‐COVID policy, respiratory pathogens did not show a noticeable increase. hMPV is mostly transmitted through respiratory droplets produced when an infected person coughs or sneezes [38]. The virus can also be contracted by contacting contaminated surfaces and then touching the face, eyes, nose, or mouth [38]. While no particular antiviral therapies or vaccinations are currently available, preventive measures such as maintaining cleanliness and treating comorbidities remain crucial in reducing hMPV infections. hMPV management is dependent on supportive policies and focused resources. Governments should prioritize financing for research on vaccines for the infectious diseases [7].

The case fatality rate (CFR) of hMPV ranges from 2.5% to 4.4%, indicating a moderate level of disease severity, particularly among pediatric patients. Although data on CFR remains limited, these figures highlight the urgency of effective patient management and underscore the need for extensive research to determine the true CFR across different age groups and populations worldwide.

## 5 Limitations

Due to resource constraints this study utilized two open‐access databases to retrieve information, which may introduce a limitation related to publication bias. Therefore, future studies need to incorporate multiple databases to generalize the findings. The prevalence of hMPV was calculated in the context of two respiratory conditions, RTIs and CAP; therefore, the findings may not be generalizable to other respiratory illnesses. The hMPV was identified in 2001; therefore, the search terms did not include articles before 2001. Additionally, the final inclusion of the studies was based on the prevalence of hMPV with RTIs and CAP, and the review of seasonal patterns and prevalent age groups was reported in those specific studies. The study excluded non‐English, gray, and other review articles. As the study variables were limited to explain the high heterogeneity among the observed studies, future studies should consider other methodological factors. Given the three available studies, CFR was calculated from heterogenous population, thus generalization of CFR is limited.

## 6 Conclusions

This systematic review and meta‐analysis provide comprehensive evidence of the global circulation and clinical burden of hMPV across all age groups, with a pooled prevalence of 6%. The findings reaffirm the virus's disproportionate impact on infant and young populations and its well‐defined seasonal patterns, most prominent in late winter and early spring. Although the CFR, ranging from 2.5% to 4.4%, indicates a moderate severity of the disease among pediatric patients, these estimates are not generalized as global CRF. The changes in hMPV transmission dynamics observed during the COVID‐19 pandemic underscore the sensitivity of respiratory pathogens to large‐scale public health interventions. Advancing global hMPV management will require strengthened surveillance systems, standardized clinical and epidemiological reporting, and sustained investment in vaccine research and development to reduce its morbidity and mortality burden, particularly among vulnerable populations. To decrease its global impact, public health officials and researchers must emphasize hMPV surveillance, which includes examining seasonal variations and transmission dynamics.

## Author Contributions

**Sumaiya Tasnim:** methodology, software, validation, formal analysis, visualization, conceptualization, data curation, writing – original draft, writing – review and editing. **Sajida Sultana Shammy:** data curation, writing – original draft, writing – review and editing, validation, software. **Najmul Haider:** conceptualization, supervision, project administration, writing – review and editing, validation, methodology. **Md. Jamal Uddin:** data curation, investigation, validation, supervision, project administration, writing – review and editing.

## Funding

The authors have nothing to report.

## Ethics Statement

This systematic review and meta‐analysis did not require ethical approval as it did not involve direct interaction with patients, humans, or animals.

## Conflicts of Interest

The authors declare no conflicts of interest.

## Transparency Statement

Md. Jamal Uddin (corresponding author) affirms that this manuscript is an honest, accurate, and transparent account of the study being reported; that no important aspects of the study have been omitted; and that any discrepancies from the study as planned (and, if relevant, registered) have been explained.

## Supporting information


Supporting File

## Data Availability

Data described in the manuscript, code book, and analytic code will be made available upon request to corresponding author (jamal-sta@sust.edu).
