# Supplementary material for: Epidemiology of Human Metapneumovirus Infection—A Systematic Review and Meta‐Analysis
Source: Health Sci Rep. 2026 Sep 13;9(9):e73234. doi: 10.1002/hsr2.73234 (PMC13572851; doi:10.1002/hsr2.73234)
Supplement: Supplementary file 1 [file HSR2-9-e73234-s001.docx]

**Table S1: PRISMA 2020 Checklist for abstract**

| **Section and Topic** | **Item #** | **Checklist item** | **Reported (Yes/No)** |
| --- | --- | --- | --- |
| **TITLE** | | |  |
| Title | 1 | Identify the report as a systematic review. | Yes |
| **BACKGROUND** | | |  |
| Objectives | 2 | Provide an explicit statement of the main objective(s) or question(s) the review addresses. | Yes |
| **METHODS** | | |  |
| Eligibility criteria | 3 | Specify the inclusion and exclusion criteria for the review. | Yes |
| Information sources | 4 | Specify the information sources (e.g. databases, registers) used to identify studies and the date when each was last searched. | Yes |
| Risk of bias | 5 | Specify the methods used to assess risk of bias in the included studies. | Yes |
| Synthesis of results | 6 | Specify the methods used to present and synthesise results. | Yes |
| **RESULTS** | | |  |
| Included studies | 7 | Give the total number of included studies and participants and summarise relevant characteristics of studies. | Yes |
| Synthesis of results | 8 | Present results for main outcomes, preferably indicating the number of included studies and participants for each. If meta-analysis was done, report the summary estimate and confidence/credible interval. If comparing groups, indicate the direction of the effect (i.e. which group is favoured). | Yes |
| **DISCUSSION** | | |  |
| Limitations of evidence | 9 | Provide a brief summary of the limitations of the evidence included in the review (e.g. study risk of bias, inconsistency, and imprecision). | N/A |
| Interpretation | 10 | Provide a general interpretation of the results and important implications. | Yes |
| **OTHER** | | |  |
| Funding | 11 | Specify the primary source of funding for the review. | N/A |
| Registration | 12 | Provide the register name and registration number. | Yes |

**Table S2**: **PRISMA 2020 checklist for manuscript**

| **Section and Topic** | **Item #** | **Checklist item** | **Location where item is reported** |
| --- | --- | --- | --- |
| **TITLE** | | |  |
| Title | 1 | Identify the report as a systematic review. | Title |
| **ABSTRACT** | | |  |
| Abstract | 2 | See the PRISMA 2020 for Abstracts checklist. | Table S1 |
| **INTRODUCTION** | | |  |
| Rationale | 3 | Describe the rationale for the review in the context of existing knowledge. | Introduction |
| Objectives | 4 | Provide an explicit statement of the objective(s) or question(s) the review addresses. | Introduction |
| **METHODS** | | |  |
| Eligibility criteria | 5 | Specify the inclusion and exclusion criteria for the review and how studies were grouped for the syntheses. | Inclusion and exclusion |
| Information sources | 6 | Specify all databases, registers, websites, organisations, reference lists and other sources searched or consulted to identify studies. Specify the date when each source was last searched or consulted. | Search strategy |
| Search strategy | 7 | Present the full search strategies for all databases, registers and websites, including any filters and limits used. | Search strategy |
| Selection process | 8 | Specify the methods used to decide whether a study met the inclusion criteria of the review, including how many reviewers screened each record and each report retrieved, whether they worked independently, and if applicable, details of automation tools used in the process. | Inclusion and exclusion criteria |
| Data collection process | 9 | Specify the methods used to collect data from reports, including how many reviewers collected data from each report, whether they worked independently, any processes for obtaining or confirming data from study investigators, and if applicable, details of automation tools used in the process. | Data extraction |
| Data items | 10a | List and define all outcomes for which data were sought. Specify whether all results that were compatible with each outcome domain in each study were sought (e.g. for all measures, time points, analyses), and if not, the methods used to decide which results to collect. | Data extraction |
|  | 10b | List and define all other variables for which data were sought (e.g. participant and intervention characteristics, funding sources). Describe any assumptions made about any missing or unclear information. | Inclusion criteria |
| Study risk of bias assessment | 11 | Specify the methods used to assess risk of bias in the included studies, including details of the tool(s) used, how many reviewers assessed each study and whether they worked independently, and if applicable, details of automation tools used in the process. | Critical appraisal and publication bias |
| Effect measures | 12 | Specify for each outcome the effect measure(s) (e.g. risk ratio, mean difference) used in the synthesis or presentation of results. | Inclusion and exclusion criteria |
| Synthesis methods | 13a | Describe the processes used to decide which studies were eligible for each synthesis (e.g. tabulating the study intervention characteristics and comparing against the planned groups for each synthesis (item #5)). | Critical appraisal and publication bias |
|  | 13b | Describe any methods required to prepare the data for presentation or synthesis, such as handling of missing summary statistics, or data conversions. | N/A |
|  | 13c | Describe any methods used to tabulate or visually display results of individual studies and syntheses. | Statistical analysis |
|  | 13d | Describe any methods used to synthesize results and provide a rationale for the choice(s). If meta-analysis was performed, describe the model(s), method(s) to identify the presence and extent of statistical heterogeneity, and software package(s) used. | Statistical analysis |
|  | 13e | Describe any methods used to explore possible causes of heterogeneity among study results (e.g. subgroup analysis, meta-regression). | Statistical analysis |
|  | 13f | Describe any sensitivity analyses conducted to assess robustness of the synthesized results. | Critical appraisal and publication bias |
| Reporting bias assessment | 14 | Describe any methods used to assess risk of bias due to missing results in a synthesis (arising from reporting biases). | N/A |
| Certainty assessment | 15 | Describe any methods used to assess certainty (or confidence) in the body of evidence for an outcome. | N/A |
| **RESULTS** | | |  |
| Study selection | 16a | Describe the results of the search and selection process, from the number of records identified in the search to the number of studies included in the review, ideally using a flow diagram. | Figure 1 |
|  | 16b | Cite studies that might appear to meet the inclusion criteria, but which were excluded, and explain why they were excluded. | Figure 1 |
| Study characteristics | 17 | Cite each included study and present its characteristics. | Table S5 |
| Risk of bias in studies | 18 | Present assessments of risk of bias for each included study. | Table S5 |
| Results of individual studies | 19 | For all outcomes, present, for each study: (a) summary statistics for each group (where appropriate) and (b) an effect estimates and its precision (e.g. confidence/credible interval), ideally using structured tables or plots. | Meta-analysis |
| Results of syntheses | 20a | For each synthesis, briefly summarise the characteristics and risk of bias among contributing studies. | Table S5 |
|  | 20b | Present results of all statistical syntheses conducted. If meta-analysis was done, present for each the summary estimate and its precision (e.g. confidence/credible interval) and measures of statistical heterogeneity. If comparing groups, describe the direction of the effect. | Meta-analysis |
|  | 20c | Present results of all investigations of possible causes of heterogeneity among study results. | Meta-analysis |
|  | 20d | Present results of all sensitivity analyses conducted to assess the robustness of the synthesized results. | Meta-analysis  (at the end) |
| Reporting biases | 21 | Present assessments of risk of bias due to missing results (arising from reporting biases) for each synthesis assessed. | N/A |
| Certainty of evidence | 22 | Present assessments of certainty (or confidence) in the body of evidence for each outcome assessed. | N/A |
| **DISCUSSION** | | |  |
| Discussion | 23a | Provide a general interpretation of the results in the context of other evidence. | Discussion |
|  | 23b | Discuss any limitations of the evidence included in the review. | Limitations |
|  | 23c | Discuss any limitations of the review processes used. | Limitations |
|  | 23d | Discuss implications of the results for practice, policy, and future research. | Conclusion |
| **OTHER INFORMATION** | | |  |
| Registration and protocol | 24a | Provide registration information for the review, including register name and registration number, or state that the review was not registered. | Methods |
|  | 24b | Indicate where the review protocol can be accessed, or state that a protocol was not prepared. | Methods |
|  | 24c | Describe and explain any amendments to information provided at registration or in the protocol. | Methods |
| Support | 25 | Describe sources of financial or non-financial support for the review, and the role of the funders or sponsors in the review. | N/A |
| Competing interests | 26 | Declare any competing interests of review authors. | Conflict of interest |
| Availability of data, code and other materials | 27 | Report which of the following are publicly available and where they can be found template data collection forms; data extracted from included studies; data used for all analyses; analytic code; any other materials used in the review. | N/A |

**Table S3.** **Inclusion and exclusion criteria**

| Criteria | Inclusion Criteria | Exclusion Criteria |
| --- | --- | --- |
| Publication period | - Articles published between 1 January 2001 and 30 April 2025 |  |
| Confirmation | - Articles with laboratory-confirmed hMPV |  |
| Population | - Patients tested for hMPV, including those with Acute Respiratory Infections (ARIs) or Community-Acquired Pneumonia (CAP) | - Patients with influenza, bronchitis, or other viral or bacterial respiratory infections. |
| Age group | - Studies including all age groups | - Studies focusing exclusively on children/youngsters or pregnant women |
| Study design | - Observational studies - Surveys - Cross sectional studies - Articles with an adequate sample Size - Full Text Available Journal Articles | - Randomized controlled trials / clinical trials - Intervention studies - Editorials, letters, and commentaries - Short communications - Case studies, reports, or case series - Theses and dissertations - Narrative literature reviews - Guidelines - Review article - Preprints - Cohort study. - Longitudinal Study - Not conducted in humans - Full text not available journal |
| Language | - Articles published in English | - Non-English language articles |
| Region | - Articles from any country or region worldwide |  |

**Table S4: Hemisphere-Specific Seasonality of Human Metapneumovirus (hMPV) Infections**

| Country | Hemisphere | Prone seasonality |
| --- | --- | --- |
| Taiwan | Northern | Spring–early Summer |
| USA | Northern | Winter–Spring (peak Spring: Mar) |
| Switzerland | Northern | Winter |
| Japan | Northern | Winter–Spring (peak early Spring: Feb–Apr) |
| Stockholm | Northern | Spring |
| China | Northern | Spring–Summer |
| Argentina | **Southern** | Late winter –Spring |
| Canada | Northern | Winter |
| Sri Lanka | Northern | Late Spring–Summer |
| India | Northern | Spring |
| Scotland | Northern | Winter |
| Argentina | **Southern** | Spring |
| Spain | Northern | Spring |
| Canada | Northern | Winter |
| Argentina | **Southern** | Late winter –Spring |
| USA | Northern | Spring |
| China | Northern | Winter |
| United Kingdom | Northern | Winter |
| South Africa | **Southern** | Autumn–Winter |
| Mexico | Northern | Autumn–Spring |
| Belgium | Northern | Winter |
| Tunisia | Northern | Spring |
| China | Northern | Spring |
| India | Northern | Peak in late Winter: Feb |
| France | Northern | Winter |
| France | Northern | Winter |
| USA | Northern | Winter–Spring |
| Taiwan | Northern | Spring |
| China | Northern | Spring–Summer |
| Jordan | Northern | Winter–Spring (peak Winter: Feb) |
| Cambodia | Northern | Rainy (Apr–Oct) – overlaps with Spring–Autumn |
| China | Northern | Winter–Spring (Nov–Mar) or Spring–Summer (Mar–Jun) |
| Kuwait | Northern | Winter |
| China | Northern | Spring |
| China | Northern | Winter–Spring |
| China | Northern | Spring (peak Spring: Apr) |
| China | Northern | Winter–Spring |
| China | Northern | Winter & Spring |
| China | Northern | Winter–Spring |
| France | Northern | Winter–Spring |
| Israel | Northern | Winter–Spring |
| United Kingdom | Northern | Winter–Spring (peak Spring: Apr) |

**Table S5: Quality assessment extracted from articles.**

| **Author’s name** | **Publication**  **Year** | **JBI Score** | **Quality** | Q1 | Q2 | Q3 | Q4 | Q5 | Q6 | Q7 | Q8 |
| --- | --- | --- | --- | --- | --- | --- | --- | --- | --- | --- | --- |
| Ren, L. et al.[40] | 2009 | 6 | Moderate quality | 1 | 1 | 1 | 1 | 0 | 0 | 1 | 1 |
| Greer, R.M., et al.[41] | 2009 | 6 | Moderate quality | 1 | 1 | 1 | 1 | 0 | 0 | 1 | 1 |
| Chan, et al.[42] | 2007 | 6 | Moderate quality | 1 | 1 | 1 | 1 | 0 | 0 | 1 | 1 |
| Ebihara T, et al.[43] | 2004 | 6 | Moderate quality | 1 | 1 | 1 | 1 | 0 | 0 | 1 | 1 |
| Kuypers J, et al.[44] | 2005 | 6 | Moderate quality | 1 | 1 | 1 | 1 | 0 | 0 | 1 | 1 |
| Williams JV et al.[45] | 2004 | 6 | Moderate quality | 1 | 1 | 1 | 1 | 3 | 0 | 1 | 1 |
| Baer G et al.[46] | 2008 | 6 | Moderate quality | 1 | 1 | 1 | 1 | 0 | 0 | 1 | 1 |
| Beneri C et al.[47] | 2009 | 6 | Moderate quality | 1 | 1 | 1 | 1 | 0 | 0 | 1 | 1 |
| Abiko C et al.[48] | 2007 | 6 | Moderate quality | 1 | 1 | 1 | 1 | 0 | 0 | 1 | 1 |
| Rafiefard F et al.[49] | 2008 | 6 | Moderate quality | 1 | 1 | 1 | 1 | 0 | 0 | 1 | 1 |
| Rujipat S et al.[50] | 2006 | 6 | Moderate quality | 1 | 1 | 1 | 1 | 0 | 0 | 1 | 1 |
| Scheltinga, S.A. et al.[51] | 2005 | 5 | Moderate quality | 1 | 0 | 1 | 1 | 0 | 0 | 1 | 1 |
| Arabpour, M, et al.[52] | 2008 | 4 | Low quality | 1 | 3 | 1 | 1 | 0 | 0 | 1 | 3 |
| Johnstone et al.[53] | 2008 | 7 | High quality | 1 | 1 | 1 | 1 | 1 | 0 | 1 | 1 |
| Falsey et al.[54] | 2006 | 7 | High quality | 1 | 1 | 1 | 1 | 1 | 0 | 1 | 1 |
| Sobrinho FLDV et al.[55] | 2021 | 8 | High quality | 1 | 1 | 1 | 1 | 1 | 1 | 1 | 1 |
| Phommasone K et al.[56] | 2022 | 6 | Moderate quality | 1 | 1 | 1 | 1 | 3 | 3 | 1 | 1 |
| Tsagarakis NJ et al.[57] | 2018 | 5 | Moderate quality | 3 | 1 | 1 | 1 | 0 | 0 | 1 | 1 |
| Williams JV et al.[58] | 2005 | 8 | High quality | 1 | 1 | 1 | 1 | 1 | 1 | 1 | 1 |
| Peiris JS et al.[59] | 2003 | 6 | Moderate quality | 1 | 1 | 1 | 1 | 3 | 3 | 1 | 1 |
| Rodriguez PE et al.[60] | 2020 | 6 | Moderate quality | 1 | 1 | 1 | 1 | 3 | 3 | 1 | 1 |
| Li L et al.[61] | 2021 | 6 | Moderate quality | 1 | 1 | 1 | 1 | 9 | 9 | 1 | 1 |
| Koul PA et al.[62] | 2022 | 8 | High quality | 1 | 1 | 1 | 1 | 1 | 1 | 1 | 1 |
| Choi SH et al.[63] | 2019 | 8 | High quality | 1 | 1 | 1 | 1 | 1 | 1 | 1 | 1 |
| Zhou F et al.[64] | 2019 | 8 | High quality | 1 | 1 | 1 | 1 | 1 | 1 | 1 | 1 |
| Boivin G et al.[65] | 2003 | 6 | Moderate quality | 1 | 1 | 1 | 1 | 0 | 0 | 1 | 1 |
| Zhang SX et al.[66] | 2009 | 6 | Moderate quality | 1 | 1 | 1 | 1 | 0 | 0 | 1 | 1 |
| McCracken JP et al.[67] | 2014 | 8 | High quality | 1 | 1 | 1 | 1 | 1 | 1 | 1 | 1 |
| Shapiro D et al.[68] | 2017 | 8 | High quality | 1 | 1 | 1 | 1 | 1 | 1 | 1 | 1 |
| Kohns Vasconcelos M et al.[69] | 2023 | 6 | Moderate quality | 1 | 1 | 1 | 1 | 3 | 0 | 1 | 1 |
| Smuts HE et al.[70] | 2004 | 8 | High quality | 1 | 1 | 1 | 1 | 1 | 1 | 1 | 1 |
| Litwin CM et al.[71] | 2013 | 6 | Moderate quality | 1 | 1 | 1 | 1 | 3 | 0 | 1 | 1 |
| Hopkins MJ et al.[72] | 2008 | 6 | Moderate quality | 1 | 1 | 1 | 1 | 3 | 0 | 1 | 1 |
| Gaunt E et al.[73] | 2009 | 6 | Moderate quality | 1 | 1 | 1 | 1 | 3 | 0 | 1 | 1 |
| Marcone DN et al.[74] | 2013 | 6 | Moderate quality | 1 | 1 | 1 | 1 | 3 | 0 | 1 | 1 |
| García-García M et al.[75] | 2006 | 8 | High quality | 1 | 1 | 1 | 1 | 1 | 1 | 1 | 1 |
| Hamano-Hasegawa K et al.[76] | 2008 | 6 | Moderate quality | 1 | 1 | 1 | 1 | 3 | 3 | 1 | 1 |
| Bharaj P et al.[77] | 2009 | 6 | Moderate quality | 1 | 1 | 1 | 1 | 3 | 3 | 1 | 1 |
| Anand M et al.[78] | 2020 | 6 | Moderate quality | 1 | 1 | 1 | 1 | 3 | 0 | 1 | 1 |
| Gurgel RQ et al.[79] | 2016 | 8 | High quality | 1 | 1 | 1 | 1 | 1 | 1 | 1 | 1 |
| Luchsinger V et al.[80] | 2013 | 6 | Moderate quality | 1 | 1 | 1 | 1 | 3 | 0 | 1 | 1 |
| Freymuth F et al.[81] | 2006 | 6 | Moderate quality | 1 | 1 | 1 | 1 | 3 | 9 | 1 | 1 |
| Pabbaraju K et al.[82] | 2007 | 6 | Moderate quality | 1 | 1 | 1 | 1 | 3 | 0 | 1 | 1 |
| Shih HI et al.[83] | 2015 | 8 | High quality | 1 | 1 | 1 | 1 | 1 | 1 | 1 | 1 |
| Velez Rueda AJ et al.[84] | 2013 | 6 | Moderate quality | 1 | 1 | 1 | 1 | 3 | 0 | 1 | 1 |
| Arden KE et al.[85] | 2006 | 6 | Moderate quality | 1 | 1 | 1 | 1 | 3 | 0 | 1 | 1 |
| Halstead DC et al.[86] | 2013 | 6 | Moderate quality | 1 | 1 | 1 | 1 | 3 | 0 | 1 | 1 |
| Zhao Y et al.[87] | 2019 | 6 | Moderate quality | 1 | 1 | 1 | 1 | 3 | 0 | 1 | 1 |
| Bonroy C et al.[88] | 2007 | 5 | Moderate quality | 1 | 1 | 1 | 1 | 0 | 0 | 1 | 3 |
| Wang SM et al.[89] | 2006 | 5 | Moderate quality | 1 | 1 | 1 | 1 | 0 | 0 | 1 | 3 |
| Bosis S et al.[90] | 2005 | 8 | High quality | 1 | 1 | 1 | 1 | 1 | 1 | 1 | 1 |
| Madhi SA et al.[91] | 2007 | 8 | High quality | 1 | 1 | 1 | 1 | 1 | 1 | 1 | 1 |
| Debur MC et al.[92] | 2010 | 6 | Moderate quality | 1 | 1 | 1 | 1 | 3 | 0 | 1 | 1 |
| Jennings LC et al.[93] | 2004 | 5 | Moderate quality | 1 | 1 | 1 | 1 | 3 | 0 | 1 | 9 |
| Wong-Chew RM et al.[94] | 2017 | 8 | High quality | 1 | 1 | 1 | 1 | 1 | 1 | 1 | 1 |
| Ramaekers K et al.[95] | 2017 | 6 | Moderate quality | 1 | 1 | 1 | 1 | 3 | 0 | 1 | 1 |
| Taktak A et al.[96] | 2023 | 6 | Moderate quality | 1 | 1 | 1 | 1 | 3 | 0 | 1 | 1 |
| [Zhang TG et al.](https://onlinelibrary.wiley.com/authored-by/Zhang/Tie%E2%80%90Gang)[97] | 2015 | 5 | Moderate quality | 1 | 1 | 1 | 1 | 3 | 0 | 1 | 3 |
| Huang X et al.[98] | 2020 | 6 | Moderate quality | 1 | 1 | 1 | 1 | 3 | 0 | 1 | 1 |
| Swamy MA et al.[99] | 2018 | 6 | Moderate quality | 1 | 1 | 1 | 1 | 0 | 0 | 1 | 1 |
| Kumar A et al.[100] | 2023 | 6 | Moderate quality | 1 | 1 | 1 | 1 | 0 | 0 | 1 | 1 |
| Chiu YT et al.[101] | 2022 | 6 | Moderate quality | 1 | 1 | 1 | 1 | 0 | 0 | 1 | 1 |
| Cho CH et al.[102] | 2014 | 6 | Moderate quality | 1 | 1 | 1 | 1 | 3 | 3 | 1 | 1 |
| Krammer M et al.[103] | 2024 | 6 | Moderate quality | 1 | 1 | 1 | 1 | 3 | 3 | 1 | 1 |
| Lee BR et al.[104] | 2019 | 8 | High quality | 1 | 1 | 1 | 1 | 1 | 1 | 1 | 1 |
| Mengelle C et al.[105] | 2014 | 8 | High quality | 1 | 1 | 1 | 1 | 1 | 1 | 1 | 1 |
| Bouvet D et al.[106] | 2015 | 5 | Moderate quality | 1 | 1 | 1 | 1 | 0 | 0 | 1 | 3 |
| Piralla A et al.[107] | 2017 | 5 | Moderate quality | 1 | 1 | 1 | 1 | 0 | 0 | 1 | 3 |
| Asner SA et al.[108] | 2015 | 8 | High quality | 1 | 1 | 1 | 1 | 1 | 1 | 1 | 1 |
| Lin WH et al.[109] | 2022 | 6 | Moderate quality | 1 | 1 | 1 | 1 | 0 | 0 | 1 | 1 |
| Toivonen L et al.[110] | 2015 | 8 | High quality | 1 | 1 | 1 | 1 | 1 | 1 | 1 | 1 |
| Liu BSc et al.[111] | 2023 | 8 | High quality | 1 | 1 | 1 | 1 | 1 | 1 | 1 | 1 |
| Gharabaghi F et al.[112] | 2011 | 6 | Moderate quality | 1 | 1 | 1 | 1 | 0 | 0 | 1 | 1 |
| Sabharwal S et al.[113] | 2021 | 6 | Moderate quality | 1 | 1 | 1 | 1 | 0 | 0 | 1 | 1 |
| Fairchok MP et al.[114] | 2010 | 7 | High quality | 1 | 1 | 1 | 1 | 3 | 1 | 1 | 1 |
| Durigon GS et al.[115] | 2015 | 7 | High quality | 1 | 1 | 1 | 1 | 3 | 1 | 1 | 1 |
| Honkinen M et al.[116] | 2012 | 7 | High quality | 1 | 1 | 1 | 1 | 3 | 1 | 1 | 1 |
| Tai C et al.[117] | 2021 | 6 | Moderate quality | 1 | 1 | 1 | 1 | 0 | 0 | 1 | 1 |
| Etemadi MR et al.[118] | 2019 | 6 | Moderate quality | 1 | 1 | 1 | 1 | 0 | 0 | 1 | 1 |
| Zhang D et al.[119] | 2014 | 6 | Moderate quality | 1 | 1 | 1 | 1 | 0 | 0 | 1 | 1 |
| Chasqueira M et al.[120] | 2018 | 8 | High quality | 1 | 1 | 1 | 1 | 1 | 1 | 1 | 1 |
| Yajima T et al.[121] | 2022 | 6 | Moderate quality | 1 | 1 | 1 | 1 | 0 | 0 | 1 | 1 |
| Qaisy LM et al.[122] | 2012 | 6 | Moderate quality | 1 | 1 | 1 | 1 | 0 | 0 | 1 | 1 |
| Furuse Y et al.[123] | 2021 | 8 | High quality | 1 | 1 | 1 | 1 | 1 | 1 | 1 | 1 |
| Arnott A et al.[124] | 2013 | 6 | Moderate quality | 1 | 1 | 1 | 1 | 0 | 0 | 1 | 1 |
| Li J et al.[125] | 2012 | 6 | Moderate quality | 1 | 1 | 1 | 1 | 0 | 0 | 1 | 1 |
| Derrar F et al.[126] | 2019 | 6 | Moderate quality | 1 | 1 | 1 | 1 | 0 | 0 | 1 | 1 |
| Al-Turab M et al.[127] | 2012 | 6 | Moderate quality | 1 | 1 | 1 | 1 | 0 | 0 | 1 | 1 |
| Zhao H et al.[128] | 2022 | 6 | Moderate quality | 1 | 1 | 1 | 1 | 0 | 0 | 1 | 1 |
| Xu MSc B et al.[129] | 2021 | 6 | Moderate quality | 1 | 1 | 1 | 1 | 0 | 0 | 1 | 1 |
| Ye H et al.[130] | 2023 | 8 | High quality | 1 | 1 | 1 | 1 | 1 | 1 | 1 | 1 |
| Cong S et al.[131] | 2022 | 8 | High quality | 1 | 1 | 1 | 1 | 1 | 1 | 1 | 1 |
| Zhu R et al.[132] | 2020 | 8 | High quality | 1 | 1 | 1 | 1 | 1 | 1 | 1 | 1 |
| Shinkai M et al.[133] | 2024 | 8 | High quality | 1 | 1 | 1 | 1 | 1 | 1 | 1 | 1 |
| Pancham K et al.[134] | 2016 | 8 | High quality | 1 | 1 | 1 | 1 | 1 | 1 | 1 | 1 |
| Groome MJ et al.[135] | 2015 | 8 | High quality | 1 | 1 | 1 | 1 | 1 | 1 | 1 | 1 |
| Jin Y et al.[136] | 2012 | 6 | Moderate quality | 1 | 1 | 1 | 1 | 0 | 0 | 1 | 1 |
| Manoha C et al.[137] | 2007 | 8 | High quality | 1 | 1 | 1 | 1 | 1 | 1 | 1 | 1 |
| Wolf MD DG et al.[138] | 2010 | 8 | High quality | 1 | 1 | 1 | 1 | 1 | 1 | 1 | 1 |

**Table S6: Search terms**

PubMed:

Search: (‘Human Metapneumovirus’ OR ‘HMPV’ OR ‘hMPV’) AND (‘case fatality rate’ OR ‘case fatality ratio’, OR ‘CFR’ OR ‘basic reproduction number’, OR ‘R0’, OR ‘seroprevalence’ OR ‘prevalence’)

ScienceDirect:

(‘Human Metapneumovirus’) AND (‘case fatality rate’ OR ‘case fatality ratio’, OR ‘CFR’ OR ‘basic reproduction number’, OR ‘R0’, OR ‘seroprevalence’ OR ‘prevalence’)

**Table S7: Adjusted random-effects meta-regression**

| **Variable** | **Coefficient** | **SE** | **z** | **p-value** | **95% CI** |
| --- | --- | --- | --- | --- | --- |
| **Study group** |  |  |  |  |  |
| RTIs vs hMPV | 0.021 | 0.032 | 0.66 | 0.507 | (−0.041, 0.083) |
| CAP vs hMPV | 0.009 | 0.037 | 0.25 | 0.804 | (−0.064, 0.082) |
| **Age group** |  |  |  |  |  |
| Children vs Adults | 0.025 | 0.018 | 1.43 | 0.154 | (−0.009, 0.060) |
| Under 5 vs Adults | 0.025 | 0.019 | 1.29 | 0.198 | (−0.013, 0.062) |
| **Continent** |  |  |  |  |  |
| Asia vs Africa* | −0.003 | 0.039 | −0.07 | 0.946 | (−0.078, 0.073) |
| Australia vs Africa* | −0.034 | 0.064 | −0.52 | 0.600 | (−0.160, 0.092) |
| Europe vs Africa* | −0.016 | 0.041 | −0.39 | 0.700 | (−0.096, 0.064) |
| North America vs Africa* | −0.019 | 0.042 | −0.45 | 0.653 | (−0.102, 0.064) |
| South America vs Africa* | 0.035 | 0.047 | 0.75 | 0.453 | (−0.057, 0.127) |
| **COVID-19 period** |  |  |  |  |  |
| Before COVID vs During/After | −0.010 | 0.031 | −0.34 | 0.737 | (−0.071, 0.050) |
| Publication year | −0.002 | 0.002 | −1.34 | 0.181 | (−0.005, 0.001) |
| **Diagnostic method** |  |  |  |  |  |
| PCR vs RT-PCR | 0.015 | 0.023 | 0.65 | 0.516 | (−0.030, 0.060) |
| Multiplex assay vs RT-PCR | −0.015 | 0.021 | −0.71 | 0.480 | (−0.056, 0.026) |
| Other/Mixed vs RT-PCR | −0.010 | 0.021 | −0.46 | 0.648 | (−0.052, 0.032) |
| Number of studies=96, Wald $\chi^{2}$(14 df) = 10.79, Overall p-value = 0.703, Residual heterogeneity [ $\chi^{2}$(81) = 4214.27, p<.001] | | | | | |

**References:**

[40]	L. Ren *et al.*, “Prevalence of human respiratory viruses in adults with acute respiratory tract infections in Beijing, 2005–2007,” *Clin. Microbiol. Infect.*, vol. 15, no. 12, pp. 1146–1153, Dec. 2009, doi: 10.1111/j.1469-0691.2009.02746.x.

[41]	“Do rhinoviruses reduce the probability of viral co-detection during acute respiratory tract infections?,” *J. Clin. Virol.*, vol. 45, no. 1, pp. 10–15, May 2009, doi: 10.1016/j.jcv.2009.03.008.

[42]	P.-C. Chan *et al.*, “Detection of Human Metapneumovirus in Hospitalized Children with Acute Respiratory Tract Infection Using Real-time RT-PCR in a Hospital in Northern Taiwan,” *J. Formos. Med. Assoc.*, vol. 106, no. 1, pp. 16–24, Jan. 2007, doi: 10.1016/S0929-6646(09)60211-4.

[43]	T. Ebihara, R. Endo, H. Kikuta, N. Ishiguro, H. Ishiko, and K. Kobayashi, “Comparison of the seroprevalence of human metapneumovirus and human respiratory syncytial virus,” *J. Med. Virol.*, vol. 72, no. 2, pp. 304–306, Feb. 2004, doi: 10.1002/jmv.10572.

[44]	J. Kuypers, N. Wright, L. Corey, and R. Morrow, “Detection and quantification of human metapneumovirus in pediatric specimens by real-time RT-PCR,” *J. Clin. Virol. Off. Publ. Pan Am. Soc. Clin. Virol.*, vol. 33, no. 4, pp. 299–305, Aug. 2005, doi: 10.1016/j.jcv.2004.11.023.

[45]	J. V. Williams *et al.*, “Human metapneumovirus and lower respiratory tract disease in otherwise healthy infants and children,” *N. Engl. J. Med.*, vol. 350, no. 5, pp. 443–450, Jan. 2004, doi: 10.1056/NEJMoa025472.

[46]	G. Baer, U. B. Schaad, and U. Heininger, “Clinical findings and unusual epidemiologic characteristics of human metapneumovirus infections in children in the region of Basel, Switzerland,” *Eur. J. Pediatr.*, vol. 167, no. 1, pp. 63–69, Jan. 2008, doi: 10.1007/s00431-007-0427-x.

[47]	C. Beneri, C. C. Ginocchio, R. Manji, and S. Sood, “Comparison of clinical features of pediatric respiratory syncytial virus and human metapneumovirus infections,” *Infect. Control Hosp. Epidemiol.*, vol. 30, no. 12, pp. 1240–1241, Dec. 2009, doi: 10.1086/648665.

[48]	C. Abiko *et al.*, “Outbreak of human metapneumovirus detected by use of the Vero E6 cell line in isolates collected in Yamagata, Japan, in 2004 and 2005,” *J. Clin. Microbiol.*, vol. 45, no. 6, pp. 1912–1919, Jun. 2007, doi: 10.1128/JCM.01251-06.

[49]	F. Rafiefard, Z. Yun, and C. Orvell, “Epidemiologic characteristics and seasonal distribution of human metapneumovirus infections in five epidemic seasons in Stockholm, Sweden, 2002-2006,” *J. Med. Virol.*, vol. 80, no. 9, pp. 1631–1638, Sep. 2008, doi: 10.1002/jmv.21242.

[50]	R. Samransamruajkit, W. Thanasugarn, N. Prapphal, A. Theamboonlers, and Y. Poovorawan, “Human metapneumovirus in infants and young children in Thailand with lower respiratory tract infections; molecular characteristics and clinical presentations,” *J. Infect.*, vol. 52, no. 4, pp. 254–263, Apr. 2006, doi: 10.1016/j.jinf.2005.07.001.

[51]	S. A. Scheltinga, K. E. Templeton, M. F. C. Beersma, and E. C. J. Claas, “Diagnosis of human metapneumovirus and rhinovirus in patients with respiratory tract infections by an internally controlled multiplex real-time RNA PCR,” *J. Clin. Virol.*, vol. 33, no. 4, pp. 306–311, Aug. 2005, doi: 10.1016/j.jcv.2004.08.021.

[52]	M. Arabpour *et al.*, “THE HIGHEST PREVALENCE OF HUMAN METAPNEUMOVIRUS IN AHWAZ CHILDREN ACCOMPANIED BY ACUTE RESPIRATORY INFECTIONS,” *Indian J. Med. Microbiol.*, vol. 26, no. 2, pp. 123–126, Apr. 2008, doi: 10.1016/S0255-0857(21)01926-5.

[53]	J. Johnstone, S. R. Majumdar, J. D. Fox, and T. J. Marrie, “Viral Infection in Adults Hospitalized With Community-Acquired Pneumonia: Prevalence, Pathogens, and Presentation,” *Chest*, vol. 134, no. 6, pp. 1141–1148, Dec. 2008, doi: 10.1378/chest.08-0888.

[54]	A. R. Falsey, M. C. Criddle, and E. E. Walsh, “Detection of respiratory syncytial virus and human metapneumovirus by reverse transcription polymerase chain reaction in adults with and without respiratory illness,” *J. Clin. Virol.*, vol. 35, no. 1, pp. 46–50, Jan. 2006, doi: 10.1016/j.jcv.2005.04.004.

[55]	F. L. D. V. Sobrinho, D. C. Aragon, and A. P. C. P. Carlotti, “Epidemiology and factors associated with the severity of viral acute lower respiratory infection in children hospitalized in Manaus, Amazonas, in 2017-2018: An observational study,” *Medicine (Baltimore)*, vol. 100, no. 18, p. e25799, May 2021, doi: 10.1097/MD.0000000000025799.

[56]	F. Piacenza *et al.*, “Interactions between patterns of multimorbidity and functional status among hospitalized older patients: a novel approach using cluster analysis and association rule mining,” *J. Transl. Med.*, vol. 22, no. 1, p. 669, Jul. 2024, doi: 10.1186/s12967-024-05444-9.

[57]	N. J. Tsagarakis, A. Sideri, P. Makridis, A. Triantafyllou, A. Stamoulakatou, and E. Papadogeorgaki, “Age-related prevalence of common upper respiratory pathogens, based on the application of the FilmArray Respiratory panel in a tertiary hospital in Greece,” *Medicine (Baltimore)*, vol. 97, no. 22, p. e10903, Jun. 2018, doi: 10.1097/MD.0000000000010903.

[58]	J. V. Williams *et al.*, “A Prospective Study Comparing Human Metapneumovirus with Other Respiratory Viruses in Adults with Hematologic Malignancies and Respiratory Tract Infections,” *J. Infect. Dis.*, vol. 192, no. 6, pp. 1061–1065, Sep. 2005, doi: 10.1086/432732.

[59]	J. S. M. Peiris *et al.*, “Children with Respiratory Disease Associated with Metapneumovirus in Hong Kong - Volume 9, Number 6—June 2003 - Emerging Infectious Diseases journal - CDC,” Jun. 2003, doi: 10.3201/eid0906.030009.

[60]	P. E. Rodriguez *et al.*, “Human Metapneumovirus: Epidemiology and genotype diversity in children and adult patients with respiratory infection in Córdoba, Argentina,” *PloS One*, vol. 15, no. 12, p. e0244093, 2020, doi: 10.1371/journal.pone.0244093.

[61]	L. Li *et al.*, “Comparison of 11 respiratory pathogens among hospitalized children before and during the COVID-19 epidemic in Shenzhen, China,” *Virol. J.*, vol. 18, no. 1, p. 202, Oct. 2021, doi: 10.1186/s12985-021-01669-y.

[62]	P. A. Koul *et al.*, “Respiratory syncytial virus among children hospitalized with severe acute respiratory infection in Kashmir, a temperate region in northern India,” *J. Glob. Health*, vol. 12, p. 04050, Jul. 2022, doi: 10.7189/jogh.12.04050.

[63]	S.-H. Choi *et al.*, “Outcomes of severe human metapneumovirus-associated community-acquired pneumonia in adults,” *J. Clin. Virol. Off. Publ. Pan Am. Soc. Clin. Virol.*, vol. 117, pp. 1–4, Aug. 2019, doi: 10.1016/j.jcv.2019.05.007.

[64]	F. Zhou *et al.*, “Disease severity and clinical outcomes of community-acquired pneumonia caused by non-influenza respiratory viruses in adults: a multicentre prospective registry study from the CAP-China Network,” *Eur. Respir. J.*, vol. 54, no. 2, p. 1802406, Aug. 2019, doi: 10.1183/13993003.02406-2018.

[65]	G. Boivin *et al.*, “Human metapneumovirus infections in hospitalized children,” *Emerg. Infect. Dis.*, vol. 9, no. 6, pp. 634–640, Jun. 2003, doi: 10.3201/eid0906.030017.

[66]	S. X. Zhang, R. Tellier, R. Zafar, R. Cheung, D. Adachi, and S. E. Richardson, “Comparison of human metapneumovirus infection with respiratory syncytial virus infection in children,” *Pediatr. Infect. Dis. J.*, vol. 28, no. 11, pp. 1022–1024, Nov. 2009, doi: 10.1097/INF.0b013e3181aa6853.

[67]	J. P. McCracken *et al.*, “Comparative epidemiology of human metapneumovirus- and respiratory syncytial virus-associated hospitalizations in Guatemala,” *Influenza Other Respir. Viruses*, vol. 8, no. 4, pp. 414–421, 2014, doi: 10.1111/irv.12251.

[68]	D. Shapiro *et al.*, “Burden and Seasonality of Viral Acute Respiratory Tract Infections among Outpatients in Southern Sri Lanka,” *Am. J. Trop. Med. Hyg.*, vol. 97, no. 1, pp. 88–96, Jul. 2017, doi: 10.4269/ajtmh.17-0032.

[69]	M. Kohns Vasconcelos *et al.*, “Detection of mostly viral pathogens and high proportion of antibiotic treatment initiation in hospitalised children with community-acquired pneumonia in Switzerland - baseline findings from the first two years of the KIDS-STEP trial,” *Swiss Med. Wkly.*, vol. 153, p. 40040, Feb. 2023, doi: 10.57187/smw.2023.40040.

[70]	H. E. Smuts, J. Kannemeyer, L. Smit, and T. Smith, “Human metapneumovirus infection in South African children hospitalised with respiratory tract disease,” *South Afr. Med. J. Suid-Afr. Tydskr. Vir Geneeskd.*, vol. 94, no. 5, pp. 359–361, May 2004.

[71]	“Seasonality and prevalence of respiratory pathogens detected by multiplex PCR at a tertiary care medical center | Archives of Virology.” Accessed: Nov. 02, 2025. [Online]. Available: https://link.springer.com/article/10.1007/s00705-013-1794-4#citeas

[72]	M. J. Hopkins *et al.*, “Detection and characterisation of human metapneumovirus from children with acute respiratory symptoms in north-west England, UK,” *J. Clin. Virol. Off. Publ. Pan Am. Soc. Clin. Virol.*, vol. 42, no. 3, pp. 273–279, Jul. 2008, doi: 10.1016/j.jcv.2008.03.011.

[73]	E. Gaunt, E. C. McWilliam-Leitch, K. Templeton, and P. Simmonds, “Incidence, molecular epidemiology and clinical presentations of human metapneumovirus; assessment of its importance as a diagnostic screening target,” *J. Clin. Virol. Off. Publ. Pan Am. Soc. Clin. Virol.*, vol. 46, no. 4, pp. 318–324, Dec. 2009, doi: 10.1016/j.jcv.2009.09.016.

[74]	D. N. Marcone *et al.*, “Viral etiology of acute respiratory infections in hospitalized and outpatient children in Buenos Aires, Argentina,” *Pediatr. Infect. Dis. J.*, vol. 32, no. 3, pp. e105-110, Mar. 2013, doi: 10.1097/INF.0b013e31827cd06f.

[75]	M. l. García-García, C. Calvo, P. Pérez-Breña, J. m. De Cea, B. Acosta, and I. Casas, “Prevalence and clinical characteristics of human metapneumovirus infections in hospitalized infants in Spain,” *Pediatr. Pulmonol.*, vol. 41, no. 9, pp. 863–871, 2006, doi: 10.1002/ppul.20456.

[76]	K. Hamano-Hasegawa *et al.*, “Comprehensive detection of causative pathogens using real-time PCR to diagnose pediatric community-acquired pneumonia,” *J. Infect. Chemother. Off. J. Jpn. Soc. Chemother.*, vol. 14, no. 6, pp. 424–432, Dec. 2008, doi: 10.1007/s10156-008-0648-6.

[77]	“Respiratory viral infections detected by multiplex PCR among pediatric patients with lower respiratory tract infections seen at an urban hospital in Delhi from 2005 to 2007 | Virology Journal | Full Text.” Accessed: Nov. 02, 2025. [Online]. Available: https://virologyj.biomedcentral.com/articles/10.1186/1743-422X-6-89

[78]	M. Anand and P. Nimmala, “Seasonal incidence of respiratory viral infections in Telangana, India: utility of a multiplex PCR assay to bridge the knowledge gap,” *Trop. Med. Int. Health TM IH*, vol. 25, no. 12, pp. 1503–1509, Dec. 2020, doi: 10.1111/tmi.13501.

[79]	R. Q. Gurgel *et al.*, “Relative frequency, Possible Risk Factors, Viral Codetection Rates, and Seasonality of Respiratory Syncytial Virus Among Children With Lower Respiratory Tract Infection in Northeastern Brazil,” *Medicine (Baltimore)*, vol. 95, no. 15, p. e3090, Apr. 2016, doi: 10.1097/MD.0000000000003090.

[80]	V. Luchsinger *et al.*, “Community-acquired pneumonia in Chile: the clinical relevance in the detection of viruses and atypical bacteria,” Nov. 2013, doi: 10.1136/thoraxjnl-2013-203551.

[81]	F. Freymuth *et al.*, “Comparison of multiplex PCR assays and conventional techniques for the diagnostic of respiratory virus infections in children admitted to hospital with an acute respiratory illness,” *J. Med. Virol.*, vol. 78, no. 11, pp. 1498–1504, Nov. 2006, doi: 10.1002/jmv.20725.

[82]	K. Pabbaraju, S. Wong, T. McMillan, B. E. Lee, and J. D. Fox, “Diagnosis and epidemiological studies of human metapneumovirus using real-time PCR,” *J. Clin. Virol. Off. Publ. Pan Am. Soc. Clin. Virol.*, vol. 40, no. 3, pp. 186–192, Nov. 2007, doi: 10.1016/j.jcv.2007.08.004.

[83]	H.-I. Shih *et al.*, “Viral Respiratory Tract Infections in Adult Patients Attending Outpatient and Emergency Departments, Taiwan, 2012-2013: A PCR/Electrospray Ionization Mass Spectrometry Study,” *Medicine (Baltimore)*, vol. 94, no. 38, p. e1545, Sep. 2015, doi: 10.1097/MD.0000000000001545.

[84]	A. J. Velez Rueda, A. S. Mistchenko, and M. Viegas, “Phylogenetic and phylodynamic analyses of human metapneumovirus in Buenos Aires (Argentina) for a three-year period (2009-2011),” *PloS One*, vol. 8, no. 4, p. e63070, 2013, doi: 10.1371/journal.pone.0063070.

[85]	K. E. Arden, P. McErlean, M. D. Nissen, T. P. Sloots, and I. M. Mackay, “Frequent detection of human rhinoviruses, paramyxoviruses, coronaviruses, and bocavirus during acute respiratory tract infections,” *J. Med. Virol.*, vol. 78, no. 9, pp. 1232–1240, Sep. 2006, doi: 10.1002/jmv.20689.

[86]	D. C. Halstead, E. R. Garcia, S. Wright, K. McKean, and P. McKitrick, “Respiratory virus identification by interval polymerase chain reaction testing in the southeastern United States,” *Am. J. Infect. Control*, vol. 41, no. 9, pp. 769–772, Sep. 2013, doi: 10.1016/j.ajic.2012.11.018.

[87]	Y. Zhao, R. Lu, J. Shen, Z. Xie, G. Liu, and W. Tan, “Comparison of viral and epidemiological profiles of hospitalized children with severe acute respiratory infection in Beijing and Shanghai, China,” *BMC Infect. Dis.*, vol. 19, no. 1, p. 729, Aug. 2019, doi: 10.1186/s12879-019-4385-5.

[88]	C. Bonroy, A. Vankeerberghen, A. Boel, and H. De Beenhouwer, “Use of a multiplex real-time PCR to study the incidence of human metapneumovirus and human respiratory syncytial virus infections during two winter seasons in a Belgian paediatric hospital,” *Clin. Microbiol. Infect. Off. Publ. Eur. Soc. Clin. Microbiol. Infect. Dis.*, vol. 13, no. 5, pp. 504–509, May 2007, doi: 10.1111/j.1469-0691.2007.01682.x.

[89]	S.-M. Wang, C.-C. Liu, H.-C. Wang, I.-J. Su, and J.-R. Wang, “Human metapneumovirus infection among children in Taiwan: a comparison of clinical manifestations with other virus-associated respiratory tract infections,” *Clin. Microbiol. Infect. Off. Publ. Eur. Soc. Clin. Microbiol. Infect. Dis.*, vol. 12, no. 12, pp. 1221–1224, Dec. 2006, doi: 10.1111/j.1469-0691.2006.01540.x.

[90]	S. Bosis, S. Esposito, H. G. M. Niesters, P. Crovari, A. D. M. E. Osterhaus, and N. Principi, “Impact of human metapneumovirus in childhood: comparison with respiratory syncytial virus and influenza viruses,” *J. Med. Virol.*, vol. 75, no. 1, pp. 101–104, Jan. 2005, doi: 10.1002/jmv.20243.

[91]	S. A. Madhi, H. Ludewick, L. Kuwanda, N. van Niekerk, C. Cutland, and K. P. Klugman, “Seasonality, incidence, and repeat human metapneumovirus lower respiratory tract infections in an area with a high prevalence of human immunodeficiency virus type-1 infection,” *Pediatr. Infect. Dis. J.*, vol. 26, no. 8, pp. 693–699, Aug. 2007, doi: 10.1097/INF.0b013e3180621192.

[92]	M. C. Debur *et al.*, “Impact of human metapneumovirus infection on in and outpatients for the years 2006-2008 in Southern Brazil,” *Mem. Inst. Oswaldo Cruz*, vol. 105, no. 8, pp. 1010–1018, Dec. 2010, doi: 10.1590/s0074-02762010000800010.

[93]	L. C. Jennings, T. P. Anderson, A. M. Werno, K. A. Beynon, and D. R. Murdoch, “Viral etiology of acute respiratory tract infections in children presenting to hospital: role of polymerase chain reaction and demonstration of multiple infections,” *Pediatr. Infect. Dis. J.*, vol. 23, no. 11, pp. 1003–1007, Nov. 2004, doi: 10.1097/01.inf.0000143648.04673.6c.

[94]	R. M. Wong-Chew *et al.*, “Respiratory viruses detected in Mexican children younger than 5 years old with community-acquired pneumonia: a national multicenter study,” *Int. J. Infect. Dis.*, vol. 62, pp. 32–38, Sep. 2017, doi: 10.1016/j.ijid.2017.06.020.

[95]	K. Ramaekers *et al.*, “Prevalence and seasonality of six respiratory viruses during five consecutive epidemic seasons in Belgium,” *J. Clin. Virol.*, vol. 94, pp. 72–78, Sep. 2017, doi: 10.1016/j.jcv.2017.07.011.

[96]	A. Taktak *et al.*, “Significant impact of COVID-19 pandemic on the circulation of respiratory viruses in Tunisia, 2020–2021,” *Clin. Epidemiol. Glob. Health*, vol. 21, p. 101306, May 2023, doi: 10.1016/j.cegh.2023.101306.

[97]	T.-G. Zhang, A.-H. Li, M. Lyu, M. Chen, F. Huang, and J. Wu, “Detection of respiratory viral and bacterial pathogens causing pediatric community-acquired pneumonia in Beijing using real-time PCR,” *Chronic Dis. Transl. Med.*, vol. 1, no. 2, pp. 110–116, 2015, doi: 10.1016/j.cdtm.2015.06.002.

[98]	X. Huang *et al.*, “Epidemiological characteristics of respiratory viruses in patients with acute respiratory infections during 2009–2018 in southern China,” *Int. J. Infect. Dis.*, vol. 98, pp. 21–32, Sep. 2020, doi: 10.1016/j.ijid.2020.06.051.

[99]	M. A. Swamy, B. Malhotra, P. Janardhan Reddy, and J. Tiwari, “Profile of Respiratory Pathogens Causing Acute Respiratory Infections in Hospitalised Children at Rajasthan a 4 Year’s Study,” *Indian J. Med. Microbiol.*, vol. 36, no. 2, pp. 163–171, Apr. 2018, doi: 10.4103/ijmm.IJMM_18_84.

[100]	A. Kumar, A. Bahal, L. Singh, S. M. Ninawe, N. Grover, and N. Suman, “Utility of multiplex real-time PCR for diagnosing paediatric acute respiratory tract infection in a tertiary care hospital,” *Med. J. Armed Forces India*, vol. 79, no. 3, pp. 286–291, May 2023, doi: 10.1016/j.mjafi.2021.05.007.

[101]	Y.-T. Chiu *et al.*, “Detection of respiratory pathogens by application of multiplex PCR panel during early period of COVID-19 pandemic in a tertiary hospital in Central Taiwan,” *J. Microbiol. Immunol. Infect.*, vol. 55, no. 6, Part 2, pp. 1144–1150, Dec. 2022, doi: 10.1016/j.jmii.2021.09.011.

[102]	C. H. Cho *et al.*, “Evaluation of the AdvanSure^TM^ real-time RT-PCR compared with culture and Seeplex RV15 for simultaneous detection of respiratory viruses,” *Diagn. Microbiol. Infect. Dis.*, vol. 79, no. 1, pp. 14–18, May 2014, doi: 10.1016/j.diagmicrobio.2014.01.016.

[103]	M. Krammer *et al.*, “Ten-year retrospective data analysis reveals frequent respiratory co-infections in hospitalized patients in Augsburg,” *iScience*, vol. 27, no. 6, p. 110136, Jun. 2024, doi: 10.1016/j.isci.2024.110136.

[104]	B. R. Lee, F. Hassan, M. A. Jackson, and R. Selvarangan, “Impact of multiplex molecular assay turn-around-time on antibiotic utilization and clinical management of hospitalized children with acute respiratory tract infections,” *J. Clin. Virol.*, vol. 110, pp. 11–16, Jan. 2019, doi: 10.1016/j.jcv.2018.11.006.

[105]	C. Mengelle *et al.*, “The use of a multiplex real-time PCR assay for diagnosing acute respiratory viral infections in children attending an emergency unit,” *J. Clin. Virol.*, vol. 61, no. 3, pp. 411–417, Nov. 2014, doi: 10.1016/j.jcv.2014.08.023.

[106]	D. Bouvet, C. Gaudy-Graffin, D. Garot, S. Sunder, C. De Gialluly, and A. Goudeau, “Diagnosis of community-acquired acute respiratory illness: From conventional microbiological methods to molecular detection (multiplex),” *Pathol. Biol.*, vol. 63, no. 2, pp. 69–73, Apr. 2015, doi: 10.1016/j.patbio.2014.12.003.

[107]	A. Piralla, B. Mariani, F. Rovida, and F. Baldanti, “Frequency of respiratory viruses among patients admitted to 26 Intensive Care Units in seven consecutive winter-spring seasons (2009–2016) in Northern Italy,” *J. Clin. Virol.*, vol. 92, pp. 48–51, Jul. 2017, doi: 10.1016/j.jcv.2017.05.004.

[108]	S. A. Asner, W. Rose, A. Petrich, S. Richardson, and D. J. Tran, “Is virus coinfection a predictor of severity in children with viral respiratory infections?,” *Clin. Microbiol. Infect.*, vol. 21, no. 3, p. 264.e1-264.e6, Mar. 2015, doi: 10.1016/j.cmi.2014.08.024.

[109]	W.-H. Lin *et al.*, “Molecular detection of respiratory pathogens in community-acquired pneumonia involving adults,” *J. Microbiol. Immunol. Infect.*, vol. 55, no. 5, pp. 829–837, Oct. 2022, doi: 10.1016/j.jmii.2021.11.009.

[110]	L. Toivonen *et al.*, “Blood MxA protein as a marker for respiratory virus infections in young children,” *J. Clin. Virol.*, vol. 62, pp. 8–13, Jan. 2015, doi: 10.1016/j.jcv.2014.11.018.

[111]	Y.-N. Liu *et al.*, “Infection and co-infection patterns of community-acquired pneumonia in patients of different ages in China from 2009 to 2020: a national surveillance study,” *Lancet Microbe*, vol. 4, no. 5, pp. e330–e339, May 2023, doi: 10.1016/S2666-5247(23)00031-9.

[112]	F. Gharabaghi, A. Hawan, S. J. Drews, and S. E. Richardson, “Evaluation of multiple commercial molecular and conventional diagnostic assays for the detection of respiratory viruses in children,” *Clin. Microbiol. Infect.*, vol. 17, no. 12, pp. 1900–1906, Dec. 2011, doi: 10.1111/j.1469-0691.2011.03529.x.

[113]	S. Sabharwal, S. Datta, C. Wattal, and A. Sachdeva, “A prospective study of prevalence of respiratory viruses causing acute respiratory infection in pediatric in-patients during pre- COVID times,” *Indian J. Med. Microbiol.*, vol. 39, no. 4, pp. 429–433, Oct. 2021, doi: 10.1016/j.ijmmb.2021.09.003.

[114]	M. P. Fairchok *et al.*, “Epidemiology of viral respiratory tract infections in a prospective cohort of infants and toddlers attending daycare,” *J. Clin. Virol.*, vol. 49, no. 1, pp. 16–20, Sep. 2010, doi: 10.1016/j.jcv.2010.06.013.

[115]	G. S. Durigon *et al.*, “Poor Outcome of Acute Respiratory Infection in Young Children with Underlying Health Condition in Brazil,” *Int. J. Infect. Dis.*, vol. 34, pp. 3–7, May 2015, doi: 10.1016/j.ijid.2015.03.003.

[116]	M. Honkinen, E. Lahti, R. Österback, O. Ruuskanen, and M. Waris, “Viruses and bacteria in sputum samples of children with community-acquired pneumonia,” *Clin. Microbiol. Infect.*, vol. 18, no. 3, pp. 300–307, Mar. 2012, doi: 10.1111/j.1469-0691.2011.03603.x.

[117]	C.-C. Tai, C.-H. Tsai, Y.-H. Huang, C.-L. Lee, H.-P. Chen, and Y.-J. Chan, “Detection of respiratory viruses in adults with respiratory tract infection using a multiplex PCR assay at a tertiary center,” *J. Microbiol. Immunol. Infect.*, vol. 54, no. 5, pp. 858–864, Oct. 2021, doi: 10.1016/j.jmii.2020.07.020.

[118]	M. R. Etemadi *et al.*, “Diversity of respiratory viruses detected among hospitalized children with acute lower respiratory tract infections at Hospital Serdang, Malaysia,” *J. Virol. Methods*, vol. 269, pp. 1–6, Jul. 2019, doi: 10.1016/j.jviromet.2019.03.013.

[119]	D. Zhang *et al.*, “Epidemiology characteristics of respiratory viruses found in children and adults with respiratory tract infections in southern China,” *Int. J. Infect. Dis.*, vol. 25, pp. 159–164, Aug. 2014, doi: 10.1016/j.ijid.2014.02.019.

[120]	M.-J. Chasqueira *et al.*, “Respiratory infections in elderly people: Viral role in a resident population of elderly care centers in Lisbon, winter 2013–2014,” *Int. J. Infect. Dis.*, vol. 69, pp. 1–7, Apr. 2018, doi: 10.1016/j.ijid.2018.01.012.

[121]	T. Yajima *et al.*, “Comparison of sputum specimens and nasopharyngeal swab specimens for diagnosis of acute human metapneumovirus-related lower respiratory tract infections in adults,” *J. Clin. Virol.*, vol. 154, p. 105238, Sep. 2022, doi: 10.1016/j.jcv.2022.105238.

[122]	L. M. Qaisy, M. M. Meqdam, A. Alkhateeb, A. Al-Shorman, H. O. AL-Rousan, and M. S. Al-Mogbel, “Human metapneumovirus in Jordan: prevalence and clinical symptoms in hospitalized pediatric patients and molecular virus characterization,” *Diagn. Microbiol. Infect. Dis.*, vol. 74, no. 3, pp. 288–291, Nov. 2012, doi: 10.1016/j.diagmicrobio.2012.07.004.

[123]	Y. Furuse *et al.*, “Epidemiological and clinical characteristics of children with acute respiratory viral infections in the Philippines: a prospective cohort study,” *Clin. Microbiol. Infect.*, vol. 27, no. 7, p. 1037.e9-1037.e14, Jul. 2021, doi: 10.1016/j.cmi.2020.09.017.

[124]	A. Arnott *et al.*, “Genetic variability of human metapneumovirus amongst an all ages population in Cambodia between 2007 and 2009,” *Infect. Genet. Evol.*, vol. 15, pp. 43–52, Apr. 2013, doi: 10.1016/j.meegid.2011.01.016.

[125]	J. Li *et al.*, “Prevalence of human metapneumovirus in adults with acute respiratory tract infection in Beijing, China,” *J. Infect.*, vol. 64, no. 1, pp. 96–103, Jan. 2012, doi: 10.1016/j.jinf.2011.10.011.

[126]	F. Derrar, K. Izri, C. Kaddache, R. Boukari, and D. Hannoun, “Virologic study of acute lower respiratory tract infections in children admitted to the paediatric department of Blida University Hospital, Algeria,” *New Microbes New Infect.*, vol. 30, p. 100536, Jul. 2019, doi: 10.1016/j.nmni.2019.100536.

[127]	M. Al-Turab, W. Chehadeh, F. Al-Mulla, and W. Al-Nakib, “Evaluation of the PrimerDesign^TM^ genesig real-time reverse transcription–polymerase chain reaction assay and the INFINITI® Respiratory Viral Panel Plus assay for the detection of human metapneumovirus in Kuwait,” *Diagn. Microbiol. Infect. Dis.*, vol. 72, no. 4, pp. 358–362, Apr. 2012, doi: 10.1016/j.diagmicrobio.2012.01.001.

[128]	H. Zhao *et al.*, “Clinical characteristics and molecular epidemiology of human metapneumovirus in children with acute lower respiratory tract infections in China, 2017 to 2019: A multicentre prospective observational study,” *Virol. Sin.*, vol. 37, no. 6, pp. 874–882, Dec. 2022, doi: 10.1016/j.virs.2022.08.007.

[129]	B. Xu *et al.*, “Seasonal association between viral causes of hospitalised acute lower respiratory infections and meteorological factors in China: a retrospective study,” *Lancet Planet. Health*, vol. 5, no. 3, pp. e154–e163, Mar. 2021, doi: 10.1016/S2542-5196(20)30297-7.

[130]	H. Ye *et al.*, “Epidemiology, genetic characteristics, and association with meteorological factors of human metapneumovirus infection in children in southern China: A 10-year retrospective study,” *Int. J. Infect. Dis.*, vol. 137, pp. 40–47, Dec. 2023, doi: 10.1016/j.ijid.2023.10.002.

[131]	S. Cong *et al.*, “Human metapneumovirus in hospitalized children with acute respiratory tract infections in Beijing, China,” *Infect. Genet. Evol.*, vol. 106, p. 105386, Dec. 2022, doi: 10.1016/j.meegid.2022.105386.

[132]	R. Zhu *et al.*, “Epidemiological and genetic characteristics of human metapneumovirus in pediatric patients across six consecutive seasons in Beijing, China,” *Int. J. Infect. Dis.*, vol. 91, pp. 137–142, Feb. 2020, doi: 10.1016/j.ijid.2019.11.012.

[133]	M. Shinkai *et al.*, “Burden of respiratory syncytial virus, human metapneumovirus and influenza virus infections in Japanese adults in the Hospitalized Acute Respiratory Tract Infection study,” *Respir. Investig.*, vol. 62, no. 4, pp. 717–725, Jul. 2024, doi: 10.1016/j.resinv.2024.05.015.

[134]	K. Pancham *et al.*, “Human Metapneumovirus Infection is Associated with Severe Respiratory Disease in Preschool Children with History of Prematurity,” *Pediatr. Neonatol.*, vol. 57, no. 1, pp. 27–34, Feb. 2016, doi: 10.1016/j.pedneo.2015.03.008.

[135]	M. J. Groome *et al.*, “Human metapneumovirus-associated severe acute respiratory illness hospitalisation in HIV-infected and HIV-uninfected South African children and adults,” *J. Clin. Virol.*, vol. 69, pp. 125–132, Aug. 2015, doi: 10.1016/j.jcv.2015.06.089.

[136]	Y. Jin *et al.*, “Newly identified respiratory viruses associated with acute lower respiratory tract infections in children in Lanzou, China, from 2006 to 2009,” *Clin. Microbiol. Infect.*, vol. 18, no. 1, pp. 74–80, Jan. 2012, doi: 10.1111/j.1469-0691.2011.03541.x.

[137]	C. Manoha, S. Espinosa, S.-L. Aho, F. Huet, and P. Pothier, “Epidemiological and clinical features of hMPV, RSV and RVs infections in young children,” *J. Clin. Virol.*, vol. 38, no. 3, pp. 221–226, Mar. 2007, doi: 10.1016/j.jcv.2006.12.005.

[138]	D. G. Wolf, D. Greenberg, Y. Shemer-Avni, N. Givon-Lavi, J. Bar-Ziv, and R. Dagan, “Association of Human Metapneumovirus with Radiologically Diagnosed Community-Acquired Alveolar Pneumonia in Young Children,” *J. Pediatr.*, vol. 156, no. 1, pp. 115–120, Jan. 2010, doi: 10.1016/j.jpeds.2009.07.014.
